# Multiple imputation of multilevel data with single-level models: A fully conditional specification approach using adjusted group means

**DOI:** 10.3758/s13428-025-02915-9

**Published:** 2026-03-02

**Authors:** Simon Grund, Oliver Lüdtke, Alexander Robitzsch

**Affiliations:** 1 University of Hamburg, Hamburg, Germany; 2 Leibniz Institute for Science and Mathematics Education, Kiel, Germany; 3 Centre for International Student Assessment, Kiel, Germany

**Keywords:** Missing data, Multiple imputation, Multilevel, Mixed-effects, Group means, Many variables

## Abstract

Missing data are a common challenge in multilevel designs, and multiple imputation (MI) is often used for handling them. Past research has shown that multilevel MI provides an effective treatment of missing data, so long as the imputation model takes the multilevel structure and the intended analyses into account, and modern methods have been developed that can accommodate even complex types of analyses. However, multilevel MI can be difficult to apply in practice, where the multilevel structure is often not very pronounced or not of immediate interest in the analysis. In these applications, existing methods can become unstable and often struggle to provide reliable results. In this article, we introduce a fully conditional specification (FCS) approach to multilevel MI that combines single-level imputation methods with group means (GM) or adjusted group means (AGM) to accommodate the multilevel structure. Based on theoretical investigations and multiple simulation studies, we evaluated the performance of these methods across balanced and unbalanced designs and with larger numbers of variables. Our findings suggest that the AGM approach – though not the GM approach – performs well across most scenarios we investigated and can even outperform conventional multilevel MI approaches in challenging applications. We also provide an illustrative example of implementing these methods in a simulated setting and discuss the implications of our findings for practice.

Missing data are a common challenge in multilevel designs, and multiple imputation (MI; Rubin, 1987) is one of the most commonly recommended methods for handling them (e.g., Schafer and Graham, 2002; see also (Enders, 2022). In MI, researchers aim to “fill in” missing data with plausible replacements generated from the observed data and an imputation model. Past research has shown that MI can be effective at handling missing data in multilevel designs, provided that the underlying imputation model adequately takes the multilevel structure and the intended analysis into account (e.g., Schafer and Yucel, 2001; Yucel, 2008; Grund et al., 2018b; Enders et al., 2016; Lüdtke et al., 2017; Enders et al., 2018; Audigier et al., 2018). This is particularly important for multilevel analyses that involve nonlinear or random effects of predictor variables with missing data, and several methods have since been developed that are specifically tailored to these applications (e.g., Grund et al., 2021; Enders et al., 2020; Erler et al., 2016, 2017; Quartagno and Carpenter, 2022; Goldstein et al., 2014; Keller and Enders, 2023). However, researchers are also often confronted with situations in which the multilevel structure is not very pronounced – or not of immediate interest – and the intended analyses are comparatively simple (e.g., McNeish et al., 2016). In these situations, it can be challenging to apply multilevel MI, because the multilevel models employed therein can be difficult to estimate or computationally unstable (e.g., Andridge, 2011, Audigier et al., 2018, Taljaard et al., 2008; see also van Buuren and Groothuis-Oudshoorn, 2011) and because practical applications often involve a larger number of variables, which have been shown to cause issues in MI more generally (Costantini et al., 2023; Grund et al., 2024; Si et al., 2023).

The present study aims to address this gap by introducing an alternative approach to multilevel MI that accommodates the multilevel structure through single-level models with group means instead of multilevel models. The purpose of this approach is to provide a simple alternative to conventional multilevel MI that remains feasible even in challenging situations. In this context, we focus on applications with relatively simple analyses for multilevel designs, such as multilevel analyses with between- and within-group effects, and single-level analyses for clustered data. The present study aims to provide a theoretical rationale for this approach and evaluate it in multiple simulation studies focused on different scenarios, including balanced and unbalanced designs as well as situations with many variables, where the multilevel structure is often not very pronounced or not of immediate interest (see also McNeish et al., 2016).

In the sections that follow, we first outline the types of multilevel analyses that we consider, followed by an overview of missing data in multilevel designs and multilevel MI. Then, we introduce group-mean-based approaches to multilevel MI and present results from four simulation studies evaluating their performance across different scenarios. Finally, we close with a discussion of our findings and their implications for research practice. All materials related to this article, including the computer code and results from the simulation studies, are available on the OSF platform (https://osf.io/mpfzu/).

## Multilevel analysis

Multilevel designs are characterized by a clustered structure in which observations are nested within higher-level observational units, for example, cross-sectional designs with persons nested in groups or longitudinal designs with repeated measurements nested in persons (e.g., Raudenbush and Bryk, 2002, Snijders and Bosker, 2012; see also Singer and Willett, 2003).

Throughout this article, we assume that the multilevel design consists of a two-level structure with persons *i* ($i = 1, \ldots , n_j$) nested in groups *j* ($j=1,\ldots ,J$), and we distinguish between balanced designs with equal-sized groups ($n_j = n$) and unbalanced designs with unequal group sizes. We are primarily interested in simple types of analyses for multilevel data, including multilevel analyses with between- and within-group effects and single-level analyses for clustered data.

### Between- and within-group effects

One of the main goals in multilevel analyses is to investigate the between-group and within-group relationships between two or more variables. To simplify the description of these analyses, we focus on the case with two variables *X* and *Y*, where *Y* is considered the outcome, and *X* is the predictor. In a general two-level model, *X* and *Y* can be decomposed as

1 $$\begin{aligned} \left[ \begin{array}{c} X_{ij} \\ Y_{ij} \end{array} \right] = \left[ \begin{array}{c} X_{j}^B \\ Y_{j}^B \end{array} \right] + \left[ \begin{array}{c} X_{ij}^W \\ Y_{ij}^W \end{array} \right] \; , \end{aligned}$$

where $X_{j}^B$ and $Y_{j}^B$ are the between-group components of *X* and *Y* in group *j*, and $X_{ij}^W$ and $Y_{ij}^W$ denote the within-group deviations of person *i* in group *j* (e.g., Rabe-Hesketh et al., 2004; Asparouhov and Muthén, 2006; Lüdtke et al., 2008; Muthén, 1994). The between- and within-group components are typically assumed to follow multivariate normal distributions

2 $$\begin{aligned} \left[ \begin{array}{c} X_{j}^B \\ Y_{j}^B \end{array} \right] \sim N(\boldsymbol{\mu }, \boldsymbol{\Sigma }_B) \; , \quad \left[ \begin{array}{c} X_{ij}^W \\ Y_{ij}^W \end{array} \right] \sim N(\textbf{0}, \boldsymbol{\Sigma }_W) \; , \end{aligned}$$

where $\boldsymbol{\mu }$ is a vector of means, $$\boldsymbol{\Sigma }_B = \left[ {\begin{smallmatrix} \tau _X^2 & \tau _\textit{XY} \\ \tau _\textit{XY} & \tau _Y^2 \end{smallmatrix}} \right] $$ is the between-group covariance matrix, and $$\boldsymbol{\Sigma }_{W} = \left[ {\begin{smallmatrix} \sigma _X^2 & \sigma _\textit{XY} \\ \sigma _\textit{XY} & \sigma _Y^2 \end{smallmatrix}} \right] $$ is the within-group covariance matrix. The between-group components $X_{j}^B$ and $Y_{j}^B$ can be understood as latent quantities that represent the differences between each group and the overall means and which correspond to the random intercepts of *X* and *Y* in a bivariate empty model (Lüdtke et al., 2008). The intraclass correlations (ICCs) of *X* and *Y* are given by $\textrm{ICC}_X = \frac{\tau _X^2}{\tau _X^2 + \sigma _X^2}$ and $\textrm{ICC}_Y = \frac{\tau _Y^2}{\tau _Y^2 + \sigma _Y^2}$, respectively, and represent the proportions of variance in *X* and *Y* that can be attributed to between-group differences (Snijders and Bosker, 2012).

Two general approaches to multilevel analyses can be distinguished, which we term the *latent* and the *manifest* covariate approach (Lüdtke et al., 2008; see also Croon and van Veldhoven, 2007, Shin and Raudenbush, 2010). In the latent covariate approach, a multilevel regression model is specified for *Y* that includes the latent between- and within-group components of *X* as predictors:

3 $$\begin{aligned} Y_{ij} = \beta _0 + \beta _\textit{YX}^W X_{ij}^W + \beta _\textit{YX}^B X_{j}^B + U_{j} + E_{ij} \; , \end{aligned}$$

where $\beta _\textit{YX}^B$ and $\beta _\textit{YX}^W$ denote the between- and within-group effects of *X* on *Y*, and $U_j$ and $E_{ij}$ denote normally distributed random intercepts and residuals, respectively. By contrast, in the manifest covariate approach, the model includes the observed (manifest) group means $\bar{X}_{\bullet j}$ and the group-mean-centered values $(X_{ij} - \bar{X}_{\bullet j})$ of *X* as predictors (Enders and Tofighi, 2007; Hofmann and Gavin, 1998; Kreft et al., 1995):

4 $$\begin{aligned} Y_{ij} = \gamma _0 + \gamma _\textit{YX}^W (X_{ij} - \bar{X}_{\bullet j}) + \gamma _\textit{YX}^B \bar{X}_{\bullet j} + U_{j} + E_{ij} \; , \end{aligned}$$

where $\gamma _\textit{YX}^B$ and $\gamma _\textit{YX}^W$ again denote the between- and within-group effects of *X* on *Y* but with the between- and within-group components of *X* now represented by the manifest group means and within-group deviations rather than their latent counterparts.

Despite their conceptual similarity, it is well known that the latent and manifest covariate models differ in terms of the estimated and population values of the between-group regression coefficients (Croon and van Veldhoven, 2007; Lüdtke et al., 2008; Grilli and Rampichini, 2011; Asparouhov and Muthén, 2006). Specifically, in a balanced multilevel design with groups of equal size $n_j = n$, the population values of the coefficients can be expressed as:

5 $$\begin{aligned} \beta _{YX}^B = \frac{\textit{Cov}(X_j^B,Y_j^B)}{\textit{Var}(X_j^B)} = \frac{\tau _{XY}}{\tau _X^2} \quad , \quad \gamma _{YX}^B = \frac{\textit{Cov}(\bar{X}_{\bullet j},Y_j^B)}{\textit{Var}(\bar{X}_{\bullet j})} = \frac{\tau _{XY} + \sigma _{XY} / n}{\tau _X^2 + \sigma _X^2 / n} \; . \end{aligned}$$

The difference between the approaches is because the manifest group means $\bar{X}_{\bullet j}$ represent only an unreliable measure of the latent between-group components, where the reliability of the group means depends on the group size $n_j$ and is given by $\textit{Rel}(\bar{X}_{\bullet j}) = \frac{\tau _X^2}{\tau _X^2 + \sigma _X^2 / n_j}$ (Raudenbush and Bryk, 2002). The between-group coefficients obtained with the manifest covariate approach will generally be lower than those in the latent covariate approach, unless the group sizes are large (for additional details, see Lüdtke et al., 2008, Asparouhov and Muthén, 2006, Croon and van Veldhoven, 2007).

### Single-level analyses

In addition to multilevel analyses, we also consider single-level analyses for clustered data, in which the relationship between *X* and *Y* is investigated without distinguishing between the between- and within-group components, namely

6 $$\begin{aligned} Y_{ij} = \beta _0 + \beta _\textit{YX} X_{ij} + E_{ij} \; , \end{aligned}$$

where $\beta _\textit{YX}$ denotes the overall effect of *X* on *Y*. In multilevel designs, estimating $\beta _\textit{YX}$ can be useful if the between- and within-group effects are not of immediate interest (McNeish et al., 2016), but statistical inferences about $\beta _\textit{YX}$ generally require corrections to the standard errors to account for the clustered data structure (e.g., cluster-robust standard errors; Cameron and Miller, 2015, Liang and Zeger, 1986).

Naturally, the analyses outlined above could be extended, for example, by including nonlinear and interaction effects of the predictors or by including random slopes of the within-group components of the predictors in multilevel analyses (e.g., Snijders and Bosker, 2012; see also Rockwood, 2020, Shin and Raudenbush, 2024). However, we do not consider these extensions in detail because they have been considered in much of the previous literature on missing data in multilevel designs, and we return to this topic in the Discussion (for additional discussion, see Enders, 2025).

## Missing data in multilevel designs

Missing data can occur for a variety of reasons, for example, when participants fail to respond to some items on a questionnaire or drop out of an ongoing study. Three types of missing data mechanisms are often distinguished (Rubin, 1987, 1976): missing completely at random (MCAR), missing at random (MAR), and missing not at random (MNAR). These mechanisms assume that there is a hypothetical complete data set that can be decomposed into an observed and a missing part $\textbf{Y} = (\textbf{Y}_\textit{obs}, \textbf{Y}_\textit{mis})$ with an indicator matrix $\textbf{R}$ that denotes which elements in $\textbf{Y}$ are observed. Under MCAR, the probability of missingness is unrelated to both the observed and the missing data, $P(\textbf{R}|\textbf{Y}) = P(\textbf{R})$. Under MAR, missingness is related to the observed but unrelated to the missing data, once the observed data are taken into account, $P(\textbf{R}|\textbf{Y}) = P(\textbf{R}|\textbf{Y}_\textit{obs})$. Finally, under MNAR, missingness is related to both the observed and the missing data, $P(\textbf{R}|\textbf{Y}) = P(\textbf{R}|\textbf{Y}_\textit{obs}, \textbf{Y}_\textit{mis})$.

In this article, we focus on MI, which requires that the missing data are either MCAR or MAR. Handling missing data under MNAR is still possible but typically requires strong assumptions about the missing data mechanism (e.g., Carpenter et al., 2023).

Multilevel designs can include missing data in many forms, for example, in person- or group-level variables and – for person-level variables – either systematically for all persons in a group or sporadically for only some of the persons (e.g., van Buuren, 2011, Resche-Rigon et al., 2013).

More generally, multilevel designs complicate the treatment of missing data using MI, because the multilevel structure must be accounted for when specifying the imputation model (e.g., Enders et al., 2016). In the following, we provide a short overview of multilevel MI before we focus on group-mean-based approaches to multilevel MI.

### Multilevel MI

The main idea of MI is to “fill in” missing values with plausible replacements that are drawn from the posterior predictive distribution of the missing data, given the observed data and an imputation model (Rubin, 1987). This process is repeated multiple times to obtain multiple completed copies of the data; the complete data are then analyzed separately, and the results are pooled to provide a final set of results. An important requirement of MI is that the imputation model must accommodate the data structure and the intended analyses (Meng, 1994). Consequently, in multilevel MI, the imputation model is often specified as a multilevel model that mimics the relevant features of the intended analyses (e.g., between- and within-group effects; Schafer and Yucel, 2002; see also Enders et al., 2016, Lüdtke et al., 2017, Goldstein et al., 2009). In the statistical literature on MI, three strategies for specifying the imputation model can be distinguished (Murray, 2018): joint modeling (JM), sequential modeling (SM), and the fully conditional specification (FCS). In this article, we focus on the FCS approach to MI because it provides the most natural basis for group-mean-based multilevel MI. The main idea of the FCS approach is to treat missing data in multiple variables by specifying a set of univariate imputation models, one for each variable with missing data, using an iterative algorithm that repeatedly steps through this sequence to generate the imputed data (Raghunathan et al., 2001; van Buuren et al., 1999, 2006).

#### FCS approach to multilevel MI

In the multilevel FCS approach, a separate imputation model is specified for each target variable with missing data, where each model must be specified in such a way that it takes the multilevel structure and intended analyses into account, and a considerable number of studies have provided recommendations for the specification of the imputation models in multilevel FCS (for an overview, see van Buuren, 2018).

For the applications considered in this article, previous research has shown that the imputation models in multilevel FCS should be specified in such a way that the model for each variable includes the other variables as well as their group means as predictors (Enders et al., 2016, 2018; Grund et al., 2018b, 2019; Mistler and Enders, 2017). Such an imputation model can be specified using either latent or manifest means, but we focus on the latter (for additional discussion, see Grund et al., 2018a).

For example, consider the multilevel analyses with between- and within-group effects, and the single-level analysis outlined above and suppose that the two variables both have missing data. Then, an imputation model for each variable could be specified that includes the other variable and its (manifest) group mean as predictors, resulting in the following pair of model equations:

7 $$\begin{aligned} \begin{aligned} X_{ij}&\!=\! \beta _{X,0} \!+\! \beta _{X,1} (Y_{ij} \!-\! \bar{Y}_{\bullet j}) + \beta _{X,2} \bar{Y}_{\bullet j} + U_{X,j} + E_{X,ij} \\ Y_{ij}&\!=\! \beta _{Y,0} \!+\! \beta _{Y,1} (X_{ij} \!-\! \bar{X}_{\bullet j}) + \beta _{Y,2} \bar{X}_{\bullet j} + U_{Y,j} + E_{Y,ij} \end{aligned} \; . \end{aligned}$$

In this model, $\beta _X$ and $\beta _Y$ denote the regression coefficients of the between- and within-group effects in the equations for *X* and *Y*, respectively, $U_{X,j}$ and $U_{Y,j}$ denote random intercepts, and $E_{X,ij}$ and $E_{Y,ij}$ denote residuals. As an alternative, the specification could be based on latent instead of manifest group means. However, previous research has indicated that these options – though not mathematically equivalent – tend to yield very similar results, except in extreme cases with very small groups and in which the group sizes are strongly unbalanced (Grund et al., 2018a; Resche-Rigon and White, 2018).

In the FCS approach, the imputation model in Eq. 7 is used to iteratively generate replacement values for each target variable, conditional on the observed and the most recently imputed values of the other variables. For example, for the missing values in *X* at iteration *t*, a new set of values $X_{\textit{mis},ij}^{(t+1)}$ would be drawn conditionally on the most recently imputed values $Y_{ij}^{(t)}$, where the group means $\bar{Y}_{\bullet j}^{(t)}$ have been updated to reflect these values (van Buuren et al., 2006; Royston, 2005). Once the $X_{\textit{mis},ij}$ have been replaced with imputed values, the same principle is applied to *Y*, iterating back and forth between the two variables.

Despite the overall efficacy of multilevel MI, previous research has also shown that the multilevel models involved in multilevel MI can sometimes be difficult to estimate, resulting in convergence problems, biased parameter estimates, and statistical inferences with poor frequentist properties (e.g., Andridge, 2011; Drechsler, 2015; Grund et al., 2018b; Enders et al., 2018). In these situations, alternatives to conventional multilevel MI are needed (see also Drechsler, 2015). These alternatives could be particularly useful in applications with many variables and in which the multilevel structure is not very pronounced (e.g., with small or heterogeneous ICCs).

## Group-mean-based approaches to multilevel MI

In the following, we describe two alternative approaches to multilevel MI, which we term the *group mean* (GM) approach and the *adjusted group mean* (AGM) approach, respectively. The general idea of these approaches is to simplify the imputation procedure by employing single-level imputation models that use group means rather than random effects to accommodate the multilevel structure.

### GM approach

In the GM approach, the specification of the imputation model incorporates the manifest group means of the variables similar to multilevel MI. However, in contrast with multilevel MI, group means are used not only to represent the between-group components of the predictor variables but also for the target variables with missing data. Specifically, with two variables *X* and *Y*, the imputation model in the GM approach becomes:

8 $$\begin{aligned} \begin{aligned} X_{ij}&\!=\! \beta _{X,0} \!+\! \beta _{X,1} (Y_{ij} \!-\! \bar{Y}_{\bullet j}) \!+\! \beta _{X,2} \bar{Y}_{\bullet j} \!+\! \beta _{X,3} \bar{X}_{\bullet j} \!+\! E_{X,ij}\\ Y_{ij}&\!=\! \beta _{Y,0} \!+\! \beta _{Y,1} (X_{ij} \!-\! \bar{X}_{\bullet j}) \!+\! \beta _{Y,2} \bar{X}_{\bullet j} \!+\! \beta _{Y,3} \bar{Y}_{\bullet j} \!+\! E_{Y,ij} \end{aligned} \; , \end{aligned}$$

where $\bar{X}_{\bullet j}$ and $\bar{Y}_{\bullet j}$ are the group means of *X* and *Y*, $\beta _X$ and $\beta _Y$ denote regression coefficients, and $E_{X,ij}$ and $E_{Y,ij}$ denote residuals as before. Notice that these imputation models are single-level rather than multilevel models, and that the group means of the target variable have been added in place of the random intercepts. These models can again be used to generate imputed values for each variable, with the main addition that these steps now also condition on the most recently imputed values of the target variables themselves. For example, for the missing values in *X* at iteration *t*, the imputed values $X_{\textit{mis},ij}^{(t+1)}$ would be drawn conditionally on the most recently imputed values $Y_{ij}^{(t)}$ and the group mean $\bar{Y}_{\bullet j}^{(t)}$ of the predictor as well as the group mean $\bar{X}_{\bullet j}^{(t)}$ of the target variable, which has been updated to reflect the most recently imputed values.

The main rationale of the GM approach is that the group means of the target variables can be regarded as a proxy for the random intercepts that would be used in multilevel MI (e.g., Raudenbush and Bryk, 2002, p.46). However, this double use of the group means is conceptually problematic because it induces a dependency in the imputation procedure, whereby each person’s imputed value on a given variable depends in part on their previously-imputed value on the same variable (i.e., values are used to “impute themselves”). For this reason, we now introduce an *adjustment* to this procedure that avoids this dependency, and we return to their theoretical properties shortly.

### AGM approach

The AGM approach follows the same idea as the GM approach but instead uses *adjusted* group means, which are computed individually for each person *i* by eliminating the person’s own value from the computation (see also Suzuki et al., 2012). Specifically, for a variable *Y*, the adjusted group mean for person *i* in group *j* is computed as

9 $$\begin{aligned} \tilde{Y}_{ij} = \frac{1}{n_j - 1} \sum _{i' \ne \, i} Y_{i'\hspace{-0.2ex}j} \; , \end{aligned}$$

where $Y_{i'\hspace{-0.2ex}j}$ refers to the values of all persons in group *j* other than person *i*. Consequently, the adjusted group means differ from the group means in that they take on different values for each person. Given the adjusted group means, the imputation models in the AGM approach are then specified as follows:

10 $$\begin{aligned} \begin{aligned} X_{ij}&\!=\! \beta _{X,0} \!+\! \beta _{X,1} (Y_{ij} \!-\! \bar{Y}_{\bullet j}) \!+\! \beta _{X,2} \bar{Y}_{\bullet j} \!+\! \beta _{X,3} \tilde{X}_{ij} \!+\! E_{X,ij} \\ Y_{ij}&\!=\! \beta _{Y,0} \!+\! \beta _{Y,1} (X_{ij} \!-\! \bar{X}_{\bullet j}) \!+\! \beta _{Y,2} \bar{X}_{\bullet j} \!+\! \beta _{Y,3} \tilde{Y}_{ij} \!+\! E_{Y,ij} \end{aligned} \; , \end{aligned}$$

Notice that the imputation model now includes the adjusted group means ($\tilde{X}_{ij}$ and $\tilde{Y}_{ij}$) of the target variables in addition to the group means of the predictors. Apart from this, the procedure for generating the imputed values in the AGM approach is similar to the GM approach. For example, for missing values in *X* at iteration *t*, the imputed values $X_{\textit{mis},ij}^{(t+1)}$ would be drawn conditionally on the most recently imputed values $Y_{ij}^{(t)}$ and the group means $\bar{Y}_{\bullet j}^{(t)}$ of the predictor as well as the adjusted group mean $\tilde{X}_{ij}^{(t)}$ of the target variable, which has been updated to reflect the most recently imputed values (excluding the person’s own value). Both the GM and the AGM approaches are implemented in the R package miceadds (Robitzsch et al., 2024).

### Rationale and theoretical properties

The GM and AGM approaches aim to simplify the imputation model by replacing multilevel models with single-level models that include (adjusted) group means to accommodate the multilevel structure. The use of group means can be justified by the fact that the group mean $\bar{Y}_{\bullet j}$ is an unbiased – though unreliable – estimator of the true between-group components with reliability equal to $\textit{Rel}(\bar{Y}_{\bullet j}) = \frac{\tau ^2}{\tau ^2 + \sigma ^2 / n_j}$, which approaches unity as the group size increases (Raudenbush and Bryk, 2002). The same justification holds for the AGM approach, which also avoids the conceptual problems of the GM approach.

**Fig. 1:**
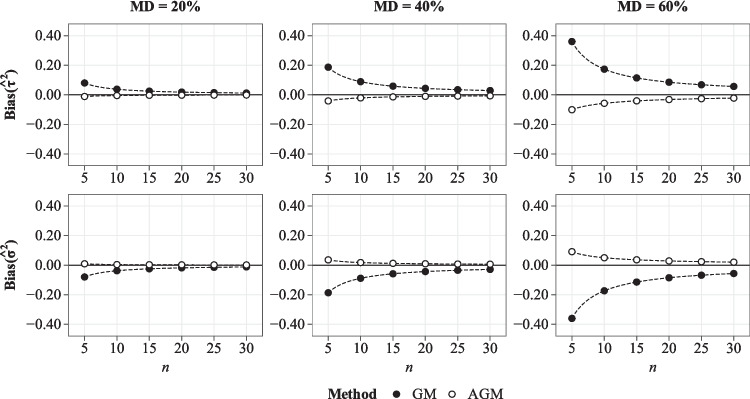
Asymptotic bias in the estimated between- and within-group variance of a single variable in the GM and AGM approach. The true variances were equal to $\tau ^2=.20$ and $\sigma ^2=.80$. *n* = group size; MD = proportion of missing data; $\tau ^2$ = between-group variance; $\sigma ^2$ = within-group variance; GM = single-level MI with group means; AGM = single-level MI with adjusted group means

The statistical properties of the GM and AGM approaches are difficult to investigate analytically due to their inherently iterative nature. To still allow us to do so, we considered a simple scenario with a single variable and a balanced design ($n_j = n$), where we focused on the bias in the between-group- ($\tau ^2$) and within-group variance ($\sigma ^2$). In addition, we assumed that the data were MCAR with a fixed proportion of missing data per group (*p*), where each group included $n_\textit{mis}$ missing and $n_\textit{obs}$ observed values, respectively. As a further simplification, we considered non-iterative versions of the GM and AGM approach, in which the group means were defined as $\bar{Y}_{\bullet j} = \frac{1}{n_\textit{obs}} \sum _{i=1}^{n_\textit{obs}} Y_{ij}$, and the adjusted group means were defined as $\tilde{Y}_{ij} = \frac{1}{n_\textit{obs} - 1} \sum _{i' \! \ne i} Y_{i'\!j}$ for cases with observed values and $\tilde{Y}_{ij} = \frac{1}{n_\textit{obs}} \sum _{i=1}^{n_\textit{obs}} Y_{ij} = \bar{Y}_{\bullet j}$ for the cases with missing values. These versions differ from how these approaches are implemented in software, but they allowed us to derive the bias in closed form. Finally, we assumed that the number of groups was large ($J \rightarrow \infty$).

The results are summarized in Fig. 1, and the derivation is given in the Appendix. Overall, the results indicated that both the GM and AGM approaches yielded biased estimates of the between- and within-group variances, but the biases differed in size and direction. For the GM approach, the bias was given by

11 $$\begin{aligned} \textit{Bias}(\hat{\tau }^2)= &  p \, \frac{n_\textit{obs} + n - 1}{(n-1) n_\textit{obs}} \, \sigma ^2 \quad \text {and} \nonumber \\ \textit{Bias}(\hat{\sigma }^2)= &  - p \, \frac{n_\textit{obs} + n - 1}{(n-1) n_\textit{obs}} \, \sigma ^2 \; , \end{aligned}$$

which is positive for $\tau ^2$ and negative for $\sigma ^2$. For the AGM approach, the bias was given by

12 $$\begin{aligned} \textit{Bias}(\hat{\tau }^2)= &  - p \, \frac{n_\textit{mis} + \frac{n_\textit{mis}-1}{n_\textit{obs}} \gamma + 1}{(n-1)(n_\textit{obs}-1)} \gamma \sigma ^2 \quad \text {and} \nonumber \\ \textit{Bias}(\hat{\sigma }^2)= &  p \, \frac{n_\textit{mis} - \gamma + 1}{(n - 1) (n_\textit{obs} - 1)} \gamma \sigma ^2 \; , \end{aligned}$$

which is negative for $\tau ^2$ and positive for $\sigma ^2$. The magnitude of the bias depends on the proportion of missing data (*p*), the group size (*n*), and the true within-group variance ($\sigma ^2$). For the AGM approach, the bias further depends on the reliability of the adjusted group means $\gamma = \frac{\tau ^2}{\tau ^2 + \sigma ^2 / (n_\textit{obs}-1)}$. For both approaches, the bias approaches zero as the group size increases ($n \rightarrow \infty$). Direct comparisons between the approaches are difficult, but the results indicated that the magnitude of the bias in the AGM approach is typically smaller and approaches zero more quickly with increasing group size as compared with the GM approach (see Fig. 1). However, given the simplified nature of the derivation, the results should only be regarded as a broad indication of their performance.

### Extension to unbalanced designs

Although the GM and AGM approaches can be applied to both balanced and unbalanced designs, previous research on multilevel MI has indicated that group-mean-based specifications of the imputation model can lead to biased parameter estimates (e.g., Grund et al., 2018a, Resche-Rigon and White, 2018; see also Carpenter et al., 2023). This is because, in unbalanced designs, the conditional distributions of the variables depend on both the group means and the group sizes $n_j$, and it has been recommended that the group sizes should be included in the imputation model (Grund et al., 2018a). For this reason, we also consider extensions of the GM and AGM approach that explicitly incorporate the group sizes in the imputation model. For the GM approach, the extended model is specified as

13 $$\begin{aligned} \begin{aligned} X_{ij}&= \beta _{X,0} + \beta _{X,1} (Y_{ij} - \bar{Y}_{\bullet j}) + \beta _{X,2} \bar{Y}_{\bullet j} + \beta _{X,3} \bar{X}_{\bullet j}\\&\quad + \beta _{X,4} w_j + \beta _{X,5} w_j \bar{Y}_{\bullet j} + \beta _{X,6} w_j \bar{X}_{\bullet j} + E_{X,ij} \\ Y_{ij}&= \beta _{Y,0} + \beta _{Y,1} (X_{ij} - \bar{X}_{\bullet j}) + \beta _{Y,2} \bar{X}_{\bullet j} + \beta _{Y,3} \bar{Y}_{\bullet j}\\&\quad + \beta _{Y,4} w_j + \beta _{Y,5} w_j \bar{X}_{\bullet j} + \beta _{Y,6} w_j \bar{Y}_{\bullet j} + E_{Y,ij} \end{aligned} \end{aligned}$$

where $w_j = f(n_j)$ represents a function of the group sizes (e.g., $w_j = 1/n_j$; see Grund et al., 2018a). The AGM approach can be extended in the same manner. The inclusion of the group sizes and their interaction with the group means effectively allows the between-group relationships between the variables to vary as a function of the group size, which has been shown previously to be effective at reducing^1^ the bias that can result from group-mean-based approaches to multilevel MI (e.g., Grund et al., 2018a; see also Resche-Rigon and White, 2018).

**Table 1:** Simulated conditions in the simulation studies

Design factor	Study 1	Study 2	Study 3a	Study 3b
Avg. group size (*n*)	5, 10, 20	10	10	10
Distribution of group sizes ($n_j$)	–	uniform (40%, 80%), bimodal (40%, 80%)	–	–
No. of groups (*J*)	50, 100, 200, 500, 1000	50, 200, 1000	50, 100, 200	50
Avg. ICC	.10, .30	.10, .30	.10, .30	.05
Range of ICCs	–	–	–	0 to .10
Between-group correlation ($\rho _B$)	.50	.50	.50	.50
Within-group correlation ($\rho _W$)	.20	.20	.50	.50
No. of variables	2	2	25	25, 35, 50
No. of variables with MD	1	1	all	all
MD mechanism	MCAR	MCAR	MCAR	MCAR
Proportion of MD	25%	25%	25%	25%
Total no. of conditions	30	24	6	3

*Note.* ICC = intraclass correlation; MD = missing data; MCAR = missing completely at random

In the sections that follow, we consider the performance of the GM and AGM approach in more detail and present the results from four simulation studies focused on different scenarios. Study 1 focused on balanced designs. Study 2 focused on unbalanced designs and whether the approaches and their extensions could accommodate unequal group sizes. Studies 3a and 3b focused on applications with many variables and applications in which the multilevel structure is not very pronounced or not of immediate interest in the analyses.

## Study 1

In Study 1, our aim was to investigate the overall performance of the GM and AGM approaches in comparison with multilevel MI in a relatively simple setting but with a relatively broad set of conditions. All files concerning the simulation, including the computer code and results, are provided on the OSF platform. In addition, we also provide an example analysis that illustrates the application of the methods with simulated data in Supplement A of the supplemental online materials and on the OSF platform.

### Data generation

Data were generated for two standardized variables *X* and *Y* from the bivariate two-level model in Eqs. 1 and 2. The between- and within-group covariance matrices $\boldsymbol{\Sigma }_B$ and $\boldsymbol{\Sigma }_W$ were specified by setting the ICCs of *X* and *Y* and their between- and within-group correlations ($\rho _B$ and $\rho _W$) to specific values. Once the complete data were generated, we induced missing data in *X* by simulating a latent response variable in accordance with the following linear model

14 $$\begin{aligned} R_{ij}^*= \alpha + \lambda Y_{ij} + \delta _{ij} \end{aligned}$$

where $\alpha$ is a quantile of the standard normal distribution that corresponds to a given probability of missing data (e.g., $\alpha = -0.67$ for 25% missing data), and $\lambda$ is a standardized regression coefficient that determines the missing data mechanism (e.g., $\lambda = 0$ for MCAR data; $\lambda \ne 0$ for MAR data), and $\delta _{ij}$ is a normally distributed residual with $\textit{Var}(\delta _{ij}) = 1 - \lambda ^2$. The values $X_{ij}$ were set to missing, when $R_{ij}^*> 0$.

#### Simulated conditions

An overview of the simulated conditions in Study 1 and the other simulation studies is shown in Table 1. In Study 1, we varied the number of persons per group ($n_j$), the number of groups (*J*), and the ICCs of the variables. Specifically, we set the number of persons per group to be equal across groups ($n_j = n$) with small, medium, and large values ($n = 5$, 10, 20) as typically encountered in cross-sectional multilevel research. The number of groups was set to values ranging from very small to very large ($J = 50$, 100, 200, 500, 1000). Finally, the ICCs were set to be equal for *X* and *Y* with small ($\textrm{ICC}_X = \textrm{ICC}_Y =.10$) and large values ($\textrm{ICC}_X = \textrm{ICC}_Y =.30$).

All other parameters were set to fixed values. Specifically, we set the between-group correlation to $\rho _B =.50$ and the within-group correlation to $\rho _W =.20$, which corresponds to a scenario with a positive contextual effect of *X* on *Y* and vice versa. For the missing data, we generated 25% MCAR data in *X*. We did not consider MAR data, because we expected that the relative differences between the imputation approaches would be similar for MCAR and MAR data^2^, and we did not vary the probability of missing data, because previous studies have shown that varying this parameter often does not materially change the results apart from scaling the differences between the different methods. In total, the simulation featured $3 \times 5 \times 2 = 30$ conditions. Each condition was replicated 1000 times.

### Imputation

The missing data were imputed with three different imputation approaches: (1) single-level FCS with the GM approach (FCS-GM), (2) single-level FCS with the AGM approach (FCS-AGM), and (3) two-level FCS (FCS-2L). To implement these procedures, we used existing imputation methods from the R packages mice (van Buuren and Groothuis-Oudshoorn, 2011) and miceadds (Robitzsch et al., 2024), and we used passive imputation steps to update the (adjusted) group means in FCS-GM and FCS-AGM. For FCS-GM and FCS-AGM, we used the norm method, and for FCS-2L, we used 2l.continuous. Each procedure was used to generate ten imputations, each after ten iterations of the FCS algorithm (Graham et al., 2007). The number of iterations was determined through visual inspections of convergence plots in five replications of a preliminary run of the simulation. In addition, we also included analyses on the basis of the complete data (CD) and listwise deletion (LD) as additional means of comparison.

### Analysis

For the analysis of the data, we considered three sets of analysis models with different parameters of interest. First, we specified an empty model with *X* as the outcome variable, where the parameter of interest was the ICC of *X*. Second, we specified a pair of two-level models based on a latent covariate approach Eq. 3 with either *X* or *Y* as the outcome and the other variable as the predictor, where the parameters of interest were the regression coefficients for the between- and within-group effects of *X* on *Y* and vice versa. Third, we specified a pair of two-level models based on the manifest covariate approach Eq. 4, where we were again interested in the between- and within-group effects. These models were considered because they allowed us to investigate different scenarios, in which (a) the variable affected by missing data was either the predictor or the outcome variable and (b) the between-group components were either represented as manifest (as in the GM and AGM approach) or latent components (as in the data-generating model). All analyses were conducted with the R package lavaan (Rosseel, 2012). The results in MI were pooled using Rubin ’s (1987) rules.

**Table 2:** Study 1: Bias of the estimated ICC of *X* ($\textrm{ICC}_{X}$)

*n*	*J*	True	CD	LD	FCS-GM	FCS-AGM	FCS-2L
Small ICCs ($\textrm{ICC}_X = \textrm{ICC}_Y = .10$)							
5	50	0.100	$-0.005$	$-0.006$	**0.100**	$\mathbf {-0.011}$	$-0.003$
	200	0.100	$-0.002$	$-0.004$	**0.106**	$-0.008$	$-0.004$
	1000	0.100	0.000	$-0.001$	**0.109**	$-0.004$	$-0.001$
10	50	0.100	$-0.005$	$-0.006$	**0.042**	$-0.008$	$-0.004$
	200	0.100	$-0.002$	$-0.002$	**0.045**	$-0.004$	$-0.002$
	1000	0.100	0.000	0.000	**0.047**	$-0.002$	0.000
20	50	0.100	$-0.003$	$-0.004$	**0.018**	$-0.005$	$-0.003$
	200	0.100	$-0.001$	$-0.001$	**0.021**	$-0.002$	$-0.001$
	1000	0.100	0.000	0.000	**0.022**	$-0.001$	0.000
Large ICCs ($\textrm{ICC}_X = \textrm{ICC}_Y = .30$)							
5	50	0.300	$-0.009$	$-0.011$	**0.070**	$-0.019$	$-0.011$
	200	0.300	$-0.001$	$-0.001$	**0.081**	$-0.007$	$-0.001$
	1000	0.300	$-0.001$	$-0.001$	**0.082**	$-0.007$	$-0.001$
10	50	0.300	$-0.012$	$-0.012$	0.023	$-0.016$	$-0.012$
	200	0.300	$-0.003$	$-0.003$	**0.033**	$-0.006$	$-0.003$
	1000	0.300	$-0.001$	0.000	**0.036**	$-0.003$	0.000
20	50	0.300	$-0.004$	$-0.004$	0.013	$-0.005$	$-0.004$
	200	0.300	$-0.001$	$-0.001$	0.016	$-0.002$	$-0.001$
	1000	0.300	$-0.001$	$-0.001$	0.016	$-0.002$	$-0.001$

*Note.* Bias values larger than ±10% of the true values are printed in bold. Monte Carlo SEs ranged from 0.000 to 0.002. *n* = group size; *J* = number of groups; CD = complete data; LD = listwise deletion; FCS-GM = single-level FCS with group means; FCS-AGM = single-level FCS with adjusted group means; FCS-2L = two-level FCS

### Evaluation criteria

For each simulated condition, missing data treatment, and parameter of interest, we evaluated the results in terms of the bias of the parameter estimates and (if applicable) the coverage rates of the 95% confidence intervals. The bias is defined as the average difference between the estimates of a parameter and its true value. The coverage rate represents the proportion of 95% confidence intervals that cover the true value. The true values that were used to compute the bias and coverage rates were derived from the population parameters except for those of the manifest covariate models, where we used the average estimates under CD in place of the true values.^3^ In addition, we report the Monte Carlo standard error for each set of results (Morris et al., 2019).

### Results

Due to the large number of results, we focus on the main findings here. Specifically, we focus on the results for the ICC of *X* and the regression coefficients of the between-group effect of *X* on *Y* in the latent covariate model, and we only summarize the remaining results. The complete set of results can be found in Supplement C of the supplemental materials and on the OSF platform.

#### Bias

**Fig. 2:**
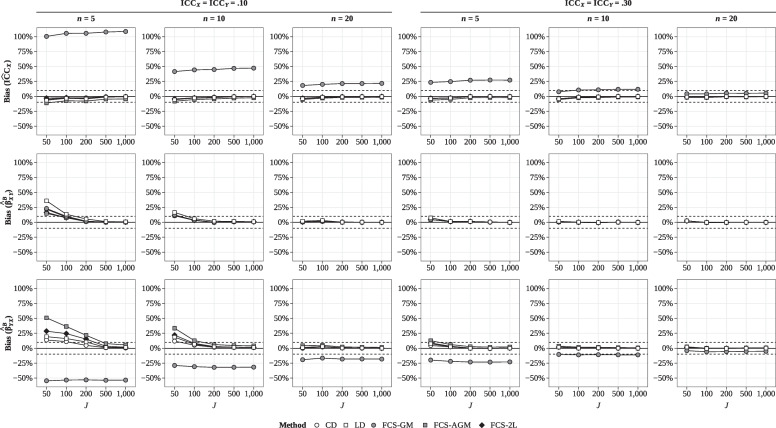
Study 1: Bias of the estimated ICC of *X* and the between-group regression coefficients in the two-level regression of *X* on *Y* ($\beta _{XY}^{B}$) and *Y* on *X* ($\beta _{XY}^{B}$, latent covariate). *n* = group size; *J* = number of groups; $\textrm{ICC}_X, \textrm{ICC}_Y$ = ICCs of *X* and *Y*; CD = complete data; LD = listwise deletion; FCS-GM = single-level FCS with group means; FCS-AGM = single-level FCS with adjusted group means; FCS-2L = two-level FCS

The results for bias of the estimated ICC of *X* are summarized in Table 2 and Fig. 2 (top row). For CD and LD, the results were essentially unbiased. By contrast, FCS-GM tended to overestimate the ICC of *X* except in conditions with larger groups ($n = 20$) and large ICCs ($\textrm{ICC}_X = \textrm{ICC}_Y =.30$). FCS-AGM showed a slight tendency for downward bias in conditions with small groups ($n=5$), but this bias was much smaller than in FCS-GM and reduced to near zero as the number of groups increased. Finally, FCS-2L provided essentially unbiased results.

For the estimated between-group effects in the latent covariate models, the results for bias are summarized in Table 3 and Fig. 2 (middle and bottom row). The results followed a different pattern depending on whether *X* was the outcome or the predictor. When *X* was the predictor ($\beta _{YX}^B$), CD and LD showed a slight upward bias in conditions with small groups ($n \le 10$) and small ICCs ($\textrm{ICC}_X = \textrm{ICC}_Y =.10$), but this bias reduced to near zero as the number of groups increased. FCS-GM generally tended to underestimate this between-group effect except in conditions with large groups ($n = 20$) and large ICCs ($\textrm{ICC}_X = \textrm{ICC}_Y =.30$). Finally, FCS-AGM and FCS-2L tended to show a slight upward bias in conditions with small groups ($n \le 10$), similar to CD and LD, which again reduced to negligible proportions as the number of groups increased. By contrast, when *X* was the outcome ($\beta _{XY}^B$), all methods performed similarly and showed a slight upward bias in the between-group effects that reduced to near zero as the number of groups increased.

For the other parameters of interest, the results can be summarized as follows (for the full results, see Supplement C). For the estimated within-group effects in the latent covariate models, we generally observed very little bias, except for FCS-GM, which tended to slightly overestimate the within-group effect of *X* on *Y* in conditions with small groups ($n=5$). For the manifest covariate models, the results followed essentially the same pattern as in the latent covariate models, except that there was no small-sample bias in the between-group effects for all methods except FCS-GM, which still exhibited the same pattern of bias.

**Table 3:** Study 1: Bias of the estimated between-group regression coefficient ($\beta _{YX}^{B}$) in the two-level regression of *Y* on *X* (latent covariate)

*n*	*J*	True	CD	LD	FCS-GM	FCS-AGM	FCS-2L
Small ICCs ($\textrm{ICC}_X = \textrm{ICC}_Y = .10$)							
5	50	0.500	**0.069**	**0.097**	$\mathbf {-0.272}$	**0.254**	**0.143**
	200	0.500	0.024	**0.053**	$\mathbf {-0.265}$	**0.108**	**0.076**
	1000	0.500	0.002	0.006	$\mathbf {-0.267}$	0.028	0.009
10	50	0.500	**0.059**	**0.093**	$\mathbf {-0.144}$	**0.167**	**0.110**
	200	0.500	0.008	0.012	$\mathbf {-0.160}$	0.031	0.014
	1000	0.500	0.006	0.007	$\mathbf {-0.158}$	0.021	0.008
20	50	0.500	0.000	0.005	$\mathbf {-0.096}$	0.022	0.007
	200	0.500	0.003	0.002	$\mathbf {-0.091}$	0.011	0.003
	1000	0.500	0.000	$-0.001$	$\mathbf {-0.091}$	0.007	0.000
Large ICCs ($\textrm{ICC}_X = \textrm{ICC}_Y = .30$)							
5	50	0.500	0.023	0.037	$\mathbf {-0.099}$	**0.063**	0.044
	200	0.500	0.001	0.000	$\mathbf {-0.115}$	0.013	0.002
	1000	0.500	0.002	0.001	$\mathbf {-0.114}$	0.011	0.001
10	50	0.500	0.010	0.009	$\mathbf {-0.052}$	0.018	0.009
	200	0.500	0.002	0.004	$\mathbf {-0.054}$	0.008	0.003
	1000	0.500	0.000	0.000	$\mathbf {-0.056}$	0.005	0.000
20	50	0.500	0.007	0.009	$-0.020$	0.012	0.008
	200	0.500	$-0.001$	$-0.001$	$-0.028$	0.001	$-0.001$
	1000	0.500	0.001	0.001	$-0.026$	0.004	0.002

*Note.* Bias values larger than ±10% of the true values are printed in bold. Monte Carlo SEs ranged from 0.001 to 0.043. *n* = group size; *J* = number of groups; CD = complete data; LD = listwise deletion; FCS-GM = single-level FCS with group means; FCS-AGM = single-level FCS with adjusted group means; FCS-2L = two-level FCS

**Table 4:** Study 1: Coverage rates (in %) of the 95% confidence intervals of the estimated between-group regression coefficient ($\beta _{YX}^{B}$) in the two-level regression of *Y* on *X* (latent covariate)

*n*	*J*	True	CD	LD	FCS-GM	FCS-AGM	FCS-2L
Small ICCs ($\textrm{ICC}_X = \textrm{ICC}_Y = .10$)							
5	50	0.500	94.3	94.1	**81.0**	96.1	95.7
	200	0.500	93.3	93.2	**38.0**	94.0	94.3
	1000	0.500	94.4	95.1	**0.1**	94.6	93.3
10	50	0.500	**91.5**	**92.3**	**89.9**	95.1	94.4
	200	0.500	95.0	94.4	**65.8**	95.2	94.6
	1000	0.500	96.4	94.5	**5.5**	94.6	94.8
20	50	0.500	94.6	95.4	92.9	94.9	95.0
	200	0.500	94.7	95.1	**83.1**	94.2	94.8
	1000	0.500	93.5	93.6	**35.7**	93.9	93.8
Large ICCs ($\textrm{ICC}_X = \textrm{ICC}_Y = .30$)							
5	50	0.500	94.0	93.5	**90.1**	93.8	93.8
	200	0.500	95.8	93.8	**73.6**	95.5	94.5
	1000	0.500	94.1	95.1	**14.8**	94.4	94.6
10	50	0.500	93.0	94.7	93.5	94.6	94.4
	200	0.500	93.5	94.7	**88**.8	94.1	94.4
	1000	0.500	94.9	94.1	**62.0**	93.6	94.5
20	50	0.500	94.6	93.6	94.3	93.6	93.9
	200	0.500	94.6	94.2	92.9	94.1	94.0
	1000	0.500	96.2	95.2	**88.6**	95.8	95.9

*Note.* Coverage rates below 92.5% are printed in bold. Monte Carlo SEs ranged from 0.1% to 1.5%. *n* = group size; *J* = number of groups; CD = complete data; LD = listwise deletion; FCS-GM = single-level FCS with group means; FCS-AGM = single-level FCS with adjusted group means; FCS-2L = two-level FCS

#### Coverage rates

For the coverage rates, we focus on the results for the between-group effects of *X* on *Y* in the latent covariate model, which are summarized in Table 4. Overall, the results followed a pattern similar to the bias. Specifically, for CD and LD, we observed coverage rates close to the nominal value of 95%, except in one condition with medium-sized groups ($n=10$), small ICCs ($\textrm{ICC}_X = \textrm{ICC}_Y =.10$), and a small number of groups. For FCS-GM, the coverage rates were often noticeably below the nominal value, especially in conditions with small groups ($n \le 10$) and small ICCs ($\textrm{ICC}_X = \textrm{ICC}_Y =.10$). FCS-AGM and FCS-2L showed near-nominal coverage rates in all conditions.

The results for the coverage rates of the other parameters also closely tracked the results for the bias and can be summarized as follows (see Supplement C). For the between-group effects of *Y* on *X* in the latent covariate model, the coverage rates for all methods were close to nominal. For the within-group effects, the coverage rates were close to nominal for all methods except FCS-GM, which showed coverage rates slightly below the nominal value in conditions with small groups ($n=5$). Finally, for the manifest covariate models, the results followed the same pattern, except for LD, which sometimes showed below-nominal coverage rates for the between-group effects.

### Summary

The results of Study 1 indicated that the AGM approach (FCS-AGM) can provide accurate results that are similar to those obtained with multilevel MI (FCS-2L) in applications with balanced designs. By contrast, the GM approach (FCS-GM) provided more biased results, and this bias often persisted in large samples with many groups. These results were also consistent with the theoretical properties of the GM and AGM approaches. Because our focus in Study 1 was on relatively simple applications with balanced data, our focus in Study 2 was on applications in unbalanced designs with unequal group sizes.

## Study 2

In Study 2, we focused on unbalanced designs and aimed to evaluate the performance of the GM and AGM approaches in applications in which the group sizes were imbalanced in different ways and to different degrees. To this end, we expanded the simulation design to include unequally-sized groups, and we included the extended versions of the GM and AGM approaches for unbalanced designs.

### Data generation

To generate the data, we adapted the procedure from Study 1 to feature unbalanced designs with unequal group sizes. Specifically, given an *average* group size *n*, we generated the group sizes $n_j$ in such a way that their distribution reflected one of two cases: a *uniform* case or a *binary* case. In the uniform case, we simulated group sizes $n_j$ that were evenly distributed in a symmetric interval around *n* (e.g., with an 80% interval around $n=10$, which would result in group sizes from 2 to 18). In the binary case, we simulated group sizes $n_j$ that were split evenly between only the extreme values of this interval (e.g., with an 80% interval around $n=10$, which would result in half of the groups having sizes of 2 and 18, respectively).

The simulated conditions that we varied included the number of groups ($J=50$, 200, 1000), the ICCs of the variables ($\textrm{ICC}_X = \textrm{ICC}_Y =.10$, .30), and the size of the interval of the group sizes around the average group size (for an overview, see Table 1).

Specifically, we set the average group size to $n=10$ and simulated group sizes in 40% and 80% intervals around this value, which we refer to as U40 and U80 in the uniform case and B40 and B80 in the binary case. The other population parameters were fixed to the same values as in Study 1. In total, the simulation featured $3 \times 2 \times 4 = 24$ conditions, each replicated 1000 times.

### Imputation and analysis

To impute the missing data, we used the same methods as in Study 1 and also included the extensions of the GM and AGM approach for unbalanced designs (henceforth called FCS-GM* and FCS-AGM*). In the extended versions (FCS-GM* and FCS-AGM*), the group sizes were incorporated in the imputation model as $w_j = 1 / n_j$ (see Eq. 13). We again used the mice and miceadds packages to implement all procedures. The analysis models were the same as in Study 1 and included the empty model with *X* as the outcome as well as the latent and manifest covariate models with *X* as the predictor and *Y* as the outcome and vice versa. The models were again fitted with the lavaan package, and we used the same evaluation criteria as before.

**Table 5:** Study 2: Bias of the estimated between-group regression coefficients ($\beta _{YX}^{B}$) in the two-level regression of *Y* on *X* (latent covariate)

$n_j$	*J*	True	CD	LD	FCS-GM	FCS-GM*	FCS-AGM	FCS-AGM*	FCS-2L
Small ICCs ($\textrm{ICC}_X = \textrm{ICC}_Y = .10$)									
U40	50	0.500	**0.057**	**0.084**	$\mathbf{-0.141}$	$\mathbf{-0.143}$	**0.176**	**0.152**	**0.119**
	200	0.500	0.008	0.012	$\mathbf{-0.161}$	$\mathbf{-0.161}$	0.035	0.030	0.015
	1000	0.500	$-0.001$	0.001	$\mathbf{-0.163}$	$\mathbf{-0.163}$	0.016	0.014	0.001
B40	50	0.500	0.019	0.037	$\mathbf{-0.157}$	$\mathbf{-0.158}$	**0.110**	**0.090**	**0.062**
	200	0.500	0.009	0.014	$\mathbf{-0.160}$	$\mathbf{-0.160}$	0.038	0.027	0.015
	1000	0.500	0.004	0.004	$\mathbf{-0.163}$	$\mathbf{-0.162}$	0.023	0.016	0.004
U80	50	0.500	0.025	0.041	$\mathbf{-0.155}$	$\mathbf{-0.165}$	**0.122**	**0.123**	**0.067**
	200	0.500	0.006	0.013	$\mathbf{-0.160}$	$\mathbf{-0.161}$	0.048	0.031	0.015
	1000	0.500	0.001	0.000	$\mathbf{-0.163}$	$\mathbf{-0.163}$	0.029	0.013	0.000
B80	50	0.500	0.021	0.044	$\mathbf{-0.151}$	$\mathbf{-0.156}$	**0.159**	**0.084**	**0.056**
	200	0.500	0.010	0.013	$\mathbf{-0.151}$	$\mathbf{-0.151}$	**0.090**	0.029	0.015
	1000	0.500	0.001	0.002	$\mathbf{-0.155}$	$\mathbf{-0.154}$	**0.070**	0.010	$-0.001$
Large ICCs ($\textrm{ICC}_X = \textrm{ICC}_Y = .30$)									
U40	50	0.500	0.015	0.018	$-0.044$	$-0.046$	0.026	0.025	0.019
	200	0.500	0.002	0.002	$\mathbf{-0.057}$	$\mathbf{-0.057}$	0.007	0.008	0.003
	1000	0.500	$-0.002$	$-0.001$	$\mathbf{-0.059}$	$\mathbf{-0.059}$	0.002	0.003	$-0.001$
B40	50	0.500	0.012	0.010	$\mathbf{-0.054}$	$\mathbf{-0.054}$	0.019	0.019	0.012
	200	0.500	0.006	0.006	$\mathbf{-0.058}$	$\mathbf{-0.058}$	0.008	0.012	0.006
	1000	0.500	0.001	0.000	$\mathbf{-0.062}$	$\mathbf{-0.062}$	0.002	0.005	0.001
U80	50	0.500	0.000	0.005	$\mathbf{-0.066}$	$\mathbf{-0.070}$	0.012	0.006	0.005
	200	0.500	0.005	0.006	$\mathbf{-0.061}$	$\mathbf{-0.060}$	0.011	0.013	0.008
	1000	0.500	0.000	0.000	$\mathbf{-0.067}$	$\mathbf{-0.066}$	0.003	0.005	0.001
B80	50	0.500	$-0.004$	0.000	$\mathbf{-0.099}$	$\mathbf{-0.098}$	0.016	0.006	0.000
	200	0.500	0.001	0.002	$\mathbf{-0.096}$	$\mathbf{-0.090}$	0.012	0.008	0.004
	1000	0.500	0.001	0.002	$\mathbf{-0.096}$	$\mathbf{-0.088}$	0.010	0.009	0.004

*Note.* Bias values larger than ±10% of the true values are printed in bold. Monte Carlo SEs ranged from 0.001 to 0.024. $n_j$ = group size distribution; U40/80 = uniform in a $40/80\%$ interval around *n*; B40/80 = bimodal with values $40/80\%$ above and below *n*; *J* = number of groups; CD = complete data; LD = listwise deletion; FCS-GM(*) = single-level FCS with group means (and group sizes); FCS-AGM(*) = single-level FCS with adjusted group means (and group sizes); FCS-2L = two-level FCS

### Results

Due to the large number of results, we focus on the results for the between-group effects in the latent covariate model and only summarize the remaining findings (for the full results, see Supplement C). Regarding the bias, the results for the between-group effects of *X* on *Y* in the latent covariate model are shown in Table 5. For CD and LD, we sometimes observed a slight upward bias, but this finding was not consistent across the types of imbalance and limited to small samples ($J=50$). For FCS-GM, we again observed biased estimates, especially in conditions with small ICCs ($\textrm{ICC}_X = \textrm{ICC}_Y =.10$). This was also true for FCS-GM*, which did not noticeably reduce bias. For FCS-AGM, we again found bias in the opposite direction of FCS-GM, but this bias again tended to be smaller and decrease further as the number of groups increased, except in the most extreme imbalance condition (B80). FCS-AGM* showed a further reduction in bias and reduced the bias to negligible proportions as the number of groups increased. Finally, for FCS-2L, the bias followed a pattern similar to FCS-AGM*. For the other parameters of interest, the results followed the same pattern as in Study 1 and were also affected by the unbalanced design in the same way (see Supplement C).

**Table 6:** Study 2: Coverage rates (in %) of the 95% confidence intervals of the estimated between-group regression coefficients ($\beta _{YX}^{B}$) in the two-level regression of *Y* on *X* (latent covariate)

$n_j$	*J*	True	CD	LD	FCS-GM	FCS-GM*	FCS-AGM	FCS-AGM*	FCS-2L
Small ICCs ($\textrm{ICC}_X = \textrm{ICC}_Y = .10$)									
U40	50	0.500	93.3	93.0	**90.5**	**90.7**	95.1	95.0	94.9
	200	0.500	93.9	93.0	**68.4**	**68.1**	94.2	94.6	93.8
	1000	0.500	95.4	95.7	**4.2**	**3.7**	94.9	95.7	95.5
B40	50	0.500	**92.2**	**91.6**	**87.5**	**86.8**	93.6	94.4	93.2
	200	0.500	94.0	93.6	**67.5**	**68.7**	93.8	93.8	93.7
	1000	0.500	95.2	94.6	**6.3**	**6.7**	93.6	93.4	93.7
U80	50	0.500	92.8	93.1	**89.6**	**89.3**	94.8	94.1	93.7
	200	0.500	94.8	93.9	**67.6**	**67.3**	93.6	94.1	93.9
	1000	0.500	95.0	94.4	**7.4**	**7.1**	93.0	94.2	94.8
B80	50	0.500	**91.8**	**92.0**	**88.2**	**87.7**	94.5	94.2	94.2
	200	0.500	94.5	93.8	**74.6**	**76.1**	92.6	94.7	94.5
	1000	0.500	95.2	94.8	**12.9**	**14.1**	**83.6**	93.8	94.4
Large ICCs ($\textrm{ICC}_X = \textrm{ICC}_Y = .30$)									
U40	50	0.500	92.8	92.5	92.7	**92.3**	92.5	93.2	92.6
	200	0.500	93.4	93.5	**87.3**	**86.9**	92.6	93.5	93.2
	1000	0.500	96.1	95.6	**60.5**	**60.4**	95.8	96.0	96.1
B40	50	0.500	93.0	93.4	92.8	93.4	93.7	94.1	94.1
	200	0.500	95.1	94.9	**88.8**	**88.8**	94.8	94.6	94.8
	1000	0.500	95.6	95.3	**56.4**	**57.6**	95.6	95.0	95.6
U80	50	0.500	94.4	93.5	93.2	92.9	94.9	93.8	93.8
	200	0.500	94.8	94.9	**89.7**	**89.8**	93.6	94.2	94.7
	1000	0.500	94.2	93.6	**53.8**	**55.3**	94.0	94.4	93.7
B80	50	0.500	94.1	93.7	**91.1**	**91.4**	92.7	93.8	93.6
	200	0.500	95.2	94.6	**81.5**	**83.2**	93.2	93.9	94.2
	1000	0.500	93.2	94.0	**31.7**	**40.2**	93.2	93.9	93.6

*Note.* Coverage rates below 92.5% are printed in bold. Monte Carlo SEs ranged from 0.6% to 1.6%. $n_j$ = group size distribution; U40/80 = uniform in a $40/80\%$ interval around *n*; B40/80 = bimodal with values $40/80\%$ above and below *n*; *J* = number of groups; CD = complete data; LD = listwise deletion; FCS-GM(*) = single-level FCS with group means (and group sizes); FCS-AGM(*) = single-level FCS with adjusted group means (and group sizes); FCS-2L = two-level FCS

Regarding the coverage rates, the results for the between-group effects of *X* on *Y* in the latent covariate model are shown in Table 6. The overall pattern of results was again similar to the bias. Specifically, for CD and LD, the coverage rates were typically close to the nominal value of 95%. For FCS-GM and FCS-GM*, we often observed coverage rates that were noticeably below the nominal value. For FCS-AGM, the coverage rates were close to the nominal value, except in one condition with extremely imbalanced groups (B80). Finally, FCS-AGM* and FCS-2L showed near-nominal coverage rates in all conditions. The results for the coverage rates for the other parameters followed the same pattern as in Study 1 and showed the same impact of the unbalanced design as the ones for the bias (see Supplement C).

### Summary

The results of Study 2 indicated that the AGM approach (FCS-AGM) can also provide results that are similar to multilevel MI (FCS-2L) in unbalanced designs. This was often true even for the unmodified version of the AGM approach, in which the imputation model did not include the group sizes, so long as the degree of imbalance was not too extreme. For extremely imbalanced group sizes, the performance of the unmodified AGM approach became worse, but the extended version of the AGM approach (FCS-AGM*) continued to provide results that were similar to multilevel MI. However, all of the results presented so far were based on scenarios with a small number of variables and a multilevel structure that is at least somewhat pronounced, in which both group-mean-based and conventional approaches to multilevel MI should be expected to perform relatively well. For this reason, in Studies 3a and 3b, we focused on more challenging scenarios with small samples, many variables, and less pronounced multilevel structures.

## Studies 3a and 3b

Both Studies 3a and 3b were concerned with more challenging scenarios but placed different emphases in terms of sample size, the number of variables, and the strength of the multilevel structure. In Study 3a, we considered scenarios with a larger number of variables, in which the aim was to conduct both multilevel and single-level analyses. In Study 3b, we considered more extreme scenarios with an even larger number of variables and a multilevel structure that was weaker and more heterogeneous.

### Data generation

The data-generating procedure was similar to Study 1 but was adapted to feature a larger number of variables with a different pattern of between-group and within-group relationships. Specifically, we simulated data for multiple standardized variables $\textbf{X} = (X_1, \ldots , X_{K})$ using a multivariate two-level model:

15 $$\begin{aligned} \textbf{X}_{j}^B \sim N(\textbf{0}, \boldsymbol{\Sigma }_B) \; , \quad \textbf{X}_{ij}^W \sim N(\textbf{0}, \boldsymbol{\Sigma }_W) \; . \end{aligned}$$

For simplicity, we assumed that the correlations between the variables were the same for all variables, and we set the between- and within-group correlations to be equal so that the multilevel structure no longer featured contextual effects. Specifically, the between- and within-group covariance matrices were defined as

16 $$\begin{aligned} \boldsymbol{\Sigma }_B= &  \left[ \begin{array}{cccc} \tau _1^2 & \rho \tau _{1} \tau _{2} & \cdots & \rho \tau _1\tau _K \\ \rho \tau _1\tau _2 & \tau _2^2 & & \rho \tau _2\tau _K \\ \vdots & & \ddots & \vdots \\ \rho \tau _1\tau _K & \rho \tau _2\tau _K & \cdots & \tau _K^2 \end{array} \right] \; \text {and} \nonumber \\ \boldsymbol{\Sigma }_W= &  \left[ \begin{array}{cccc} \sigma _1^2 & \rho \sigma _{1} \sigma _{2} & \cdots & \rho \sigma _1\sigma _K \\ \rho \sigma _1\sigma _2 & \sigma _2^2 & & \rho \sigma _2\sigma _K \\ \vdots & & \ddots & \vdots \\ \rho \sigma _1\sigma _K & \rho \sigma _2\sigma _K & \cdots & \sigma _K^2 \end{array} \right] \; , \end{aligned}$$

where the $\tau ^2$ and the $\sigma ^2$ are the between- and within-group variances of the variables. Once the data were generated, we simulated the missing data as before. However, in contrast to the previous studies, we generated missing data on *all* variables, where each variable had the same probability of missing data.

#### Simulated conditions

The simulated conditions in Studies 3a and 3b had different emphases and are summarized in Table 1. In Study 3a, we fixed the group sizes to $n=10$, the number of variables to $K=25$, the between- and within-group correlation to $\rho =.50$, and the proportion of missing data per variable to 25% in accordance with an MCAR mechanism. The factors we varied were the number of groups (*J* = 50, 100, 200) and the ICCs, whose values we set to be homogeneous across variables ($\textrm{ICC}_{X_1} = \ldots = \textrm{ICC}_{X_{25}}$ = .10, .30). These values were chosen to mimic applications in which the main challenge is that the number of variables is relatively large in comparison with the sample size. In Study 3b, we then varied the number of variables from moderate to very large (*K* = 25, 35, 50) and specified fixed values for the ICCs, which we set to be heterogeneous in a range from 0 to .10. To this end, we divided the variables into five groups of five (for $K=25$), seven ($K=35$), or ten ($K=50$) variables, respectively, and set the ICCs of the variables in each group to .10, .075, .05, .025, and 0, respectively. The number of groups was set to $J=50$, and the other factors were set to the same values as in Study 3a. These values were chosen because they reflect the conditions encountered in practice, where the number of variables is often relatively large, and the ICCs of the variables are often heterogeneous e.g.,, (Hedges and Hedberg, 2007; Brunner et al., 2018). Study 3a thus featured $3 \times 2 = 6$ conditions, and Study 3b featured three conditions. Each condition was replicated 1000 times.

### Imputation and analysis

In Studies 3a and 3b, we used the same imputation procedures as in Study 1 (FCS-GM, FCS-AGM, and FCS-2L). To implement them, we again used the mice and miceadds packages, but we increased the number of iterations to 20. We also included CD but did not include LD due to the large proportion of cases with missing data. For the analysis of the data, we then considered five analysis models. First, we specified an empty model with $X_1$ as the outcome variable, where the parameter of interest was the ICC of $X_1$ ($\textrm{ICC}_{X_1}$). The second and third models were the latent and manifest covariate models with $X_1$ as the outcome and $X_2$ as the predictor. The fourth model was a single-level regression model with $X_1$ as the outcome and $X_2$ as the predictor. Finally, the fifth model was a single-level regression model with $X_1$ as the outcome and all other variables ($X_2, \ldots , X_{K}$) as predictors. All analyses were conducted using lavaan. For the single-level models, we used cluster-robust standard errors to account for the multilevel structure (Cameron and Miller, 2015).

**Table 7:** Study 3a: Bias and coverage rates (in %) of the 95% confidence intervals of the estimated regression coefficients ($\beta _{X_1 X_2}$) in the single- level regressions of $X_1$ on $X_2$ and $X_1$ on $X_2, \ldots , X_{25}$ (cluster-robust SEs) and the estimated between-group coefficient ($\beta _{X_1 X_2}^{B}$) in the two-level regression of $X_1$ on $X_2$ (latent covariate)

		Bias				Coverage (%)			
*J*	True	CD	FCS-GM	FCS-AGM	FCS-2L	CD	FCS-GM	FCS-AGM	FCS-2L
Small ICCs ($\textrm{ICC}_{X_1} = \ldots = \textrm{ICC}_{X_{25}} = .10$)									
*Single-level regression:* $X_1$ *on* $X_2$ ($\beta _{X_1 X_2}$)									
50	0.500	0.001	$-0.016$	$-0.016$	$-0.047$	94.6	94.9	94.4	97.1
200	0.500	0.000	$-0.005$	$-0.004$	$-0.007$	94.6	95.0	94.4	96.8
1000	0.500	$-0.000$	$-0.002$	$-0.001$	$-0.002$	95.7	94.5	94.4	97.2
*Single-level regression:* $X_1$ *on* $X_2, \ldots , X_{25}$ ($\beta _{X_1 X_2}$)									
50	0.040	0.002	0.003	0.004	0.003	92.8	94.0	95.6	98.2
200	0.040	0.000	0.000	0.000	0.000	93.8	94.0	94.1	94.4
1000	0.040	$-0.000$	$-0.001$	$-0.001$	$-0.001$	94.4	93.8	94.2	94.5
*Two-level regression (latent covariate):* $X_1$ *on* $X_2$ ($\beta _{X_1 X_2}^B$)									
50	0.500	$-0.003$	$\mathbf{-0.125}$	$-0.036$	$\mathbf{-0.132}$	95.3	96.1	97.5	99.3
200	0.500	$-0.001$	$\mathbf{-0.109}$	0.007	$-0.018$	95.4	**89.6**	95.5	98.3
1000	0.500	0.001	$\mathbf{-0.105}$	0.008	$-0.003$	95.9	**47.5**	95.0	97.3
Large ICCs ($\textrm{ICC}_{X_1} = \ldots = \textrm{ICC}_{X_{25}} = .30$)									
*Single-level regression:* $X_1$ on $X_2$ ($\beta _{X_1 X_2}$)									
50	0.500	0.000	$-0.015$	$-0.015$	$\mathbf{-0.051}$	94.2	94.3	94.1	96.5
200	0.500	0.001	$-0.003$	$-0.003$	$-0.007$	93.1	93.7	93.7	96.1
1000	0.500	0.000	$-0.001$	$-0.001$	$-0.001$	94.8	96.1	95.1	97.1
*Single-level regression:* $X_1$ on $X_2, \ldots , X_{25}$ ($\beta _{X_1 X_2}$)									
50	0.040	$-0.000$	$-0.001$	$-0.001$	$-0.001$	**90.5**	93.0	93.8	98.4
200	0.040	0.001	0.001	0.002	0.001	93.9	95.1	94.9	95.7
1000	0.040	0.000	$-0.000$	$-0.000$	$-0.000$	94.7	95.3	94.8	95.1
*Two-level regression (latent covariate):* $X_1$ *on* $X_2$ ($\beta _{X_1 X_2}^B$)									
50	0.500	0.001	$-0.036$	$-0.003$	$-0.047$	93.8	95.1	95.4	99.1
200	0.500	$-0.000$	$-0.033$	0.002	$-0.006$	94.2	93.2	95.1	97.3
1000	0.500	0.000	$-0.032$	0.003	$-0.001$	95.3	**86.2**	94.4	96.4

*Note.* Bias values larger than ±10% of the true values and coverage rates below 92.5% are printed in bold. Coverage rates above 97.5% are underlined. Monte Carlo SEs ranged from 0.000 to 0.010 for the bias and from 0.3% to 1.6% for the coverage rates. *J* = number of groups; CD = complete data; FCS-GM = single-level FCS with group means; FCS-AGM = single-level FCS with adjusted group means; FCS-2L = two-level FCS

### Results: Study 3a

Due to the large number of results in Study 3a, we focus on the results for the regression coefficients in the single-level regression models and the between-group effects in the latent covariate model, which are summarized in Table 7 (for the full results, see Supplement C). For the single-level analyses, all methods provided estimates with little or no bias, except for FCS-2L, which exhibited a slight downward bias in conditions with large ICCs (ICCs = .30) and a small number of groups ($J=50$). The coverage rates in the single-level analyses were close to the nominal value of 95% for all methods except CD, which showed coverage rates slightly below the nominal value in conditions with large ICCs (ICCs = .30) and a small number of groups ($J=50$), and FCS-2L, which showed above-nominal coverage in the same conditions and regardless of the ICCs. For the between-group coefficients in the latent covariate model, CD and FCS-AGM yielded approximately unbiased estimates and near-nominal coverage in all conditions. For FCS-GM, we observed a downward bias in the between-group effects and below-nominal coverage in conditions with small ICCs (ICCs = .10). Finally, for FCS-2L, we found little to no bias, except in one condition with small ICCs (ICCs = .10) and a small number of groups ($J=50$), but we again observed above-nominal coverage in conditions with a small number of groups ($J=50$) regardless of the ICC.

**Table 8:** Study 3b: Bias and coverage rates (in %) of the 95% confidence intervals of the estimated regression coefficients ($\beta _{X_1 X_2}$) in the single- level regressions of $X_1$ on $X_2$ and $X_1$ on $X_2, \ldots , X_{K}$ (cluster-robust SEs) and the estimated between-group coefficient ($\beta _{X_1 X_2}^{B}$) in the two-level regression of $X_1$ on $X_2$ (latent covariate)

		Bias				Coverage (%)			
*K*	True	CD	FCS-GM	FCS-AGM	FCS-2L	CD	FCS-GM	FCS-AGM	FCS-2L
*Single-level regression:* $X_1$ *on* $X_2$ ($\beta _{X_1 X_2}$)									
25	0.500	0.003	$-0.016$	$-0.015$	$\mathbf{-0.051}$	94.9	93.9	94.4	96.1
35	0.500	0.002	$-0.023$	$-0.020$	$\mathbf{-0.099}$	94.0	93.6	94.8	**91.9**
50	0.500	$-0.000$	$-0.040$	$-0.035$		95.5	**89.6**	92.9	
*Single-level regression:* $X_1$ *on* $X_2, \ldots , X_{K}$ ($\beta _{X_1 X_2}$)									
25	0.040	**0.017**	**0.015**	**0.016**	**0.010**	**90.8**	94.1	93.8	98.4
35	0.029	**0.015**	**0.017**	**0.016**	**0.008**	**91.8**	93.9	94.6	100.0
50	0.020	**0.008**	**0.005**	**0.006**		92.6	95.6	97.1	
*Two-level regression (latent covariate):* $X_1$ *on* $X_2$ ($\beta _{X_1 X_2}^B$)									
25	0.500	0.014	$\mathbf{-0.113}$	$-0.018$	$\mathbf{-0.125}$	92.8	94.8	95.5	99.3
35	0.500	$-0.001$	$\mathbf{-0.123}$	$\mathbf{-0.060}$	$\mathbf{-0.240}$	**92.0**	94.5	96.4	100.0
50	0.500	$-0.010$	$\mathbf{-0.150}$	$\mathbf{-0.112}$		93.7	94.9	96.0	

*Note.* Bias values larger than ±10% of the true values and coverage rates below 92.5% are printed in bold. Coverage rates above 97.5% are underlined. Monte Carlo SEs ranged from 0.001 to 0.012 for the bias and from 0.0% to 1.0% for the coverage rates. *K* = number of variables; CD = complete data; FCS-GM = single-level FCS with group means; FCS-AGM = single-level FCS with adjusted group means; FCS-2L = two-level FCS

### Results: Study 3b

For the results of Study 3b, we again focus on the main findings, which are shown in Table 8 (for the full results, see Supplement C). No results are shown for FCS-2L for the condition with $K=50$ variables, because FCS-2L failed consistently in this condition.^4^ By contrast, CD and FCS-GM provided results in all replications and all conditions, and FCS-AGM also did so except in the condition with $K=50$, where it failed in four (0.4%) replications. In the single-level regression of $X_1$ on $X_2$, CD and FCS-AGM provided results with little bias and near-nominal coverage rates of the 95% confidence intervals. For FCS-GM, the bias was also low, but the coverage was reduced in conditions with many variables ($K=50$). For FCS-2L, the results were biased in all conditions, and coverage was reduced unless the number of variables was only moderate ($K=25$). In the single-level regression of $X_1$ on all other variables, the results were biased with poor coverage for most methods, including CD. Specifically, CD tended to overestimate the regression coefficient with below-nominal coverage rates of the 95% confidence intervals, particularly when the number of variables was only moderate ($K=25$, 35). The results for FCS-GM and FCS-AGM were typically close to CD but with higher (near-nominal) coverage rates. For FCS-2L, the results showed nominally less bias, but the parameter estimates were less similar to CD, and coverage was too high in all conditions. Finally, in the two-level regression of $X_1$ on $X_2$, CD provided results with little bias and near-nominal coverage, whereas the other methods tended to provide poorer results, especially in conditions with many variables ($K=35$, 50). For FCS-GM, the results were noticeably biased but with nominal coverage. FCS-AGM also showed bias with nominal coverage, but the bias was the lowest among the MI procedures and limited to conditions with many variables ($K=35$, 50). Finally, FCS-2L showed the strongest bias and above-nominal coverage.

### Summary

The results of Studies 3a and 3b indicated that the AGM approach (FCS-AGM) can sometimes provide more accurate results than multilevel MI (FCS-2L) in applications with many variables and a multilevel structure that is not very pronounced. This difference was smaller in Study 3a, where the number of variables was still moderate but were more noticeable in Study 3b, where the number of variables approached the number of groups, and the ICCs were small and heterogeneous. In these situations, the AGM approach tended to perform better than multilevel MI and showed acceptable performance even when multilevel MI could not be used at all.

## Discussion

In the present article, we introduced the GM and AGM approach to multilevel MI, which combines single-level imputation methods with (adjusted) group means to accommodate the multilevel structure. After investigating the theoretical properties of the GM and AGM approach, we evaluated them in three simulation studies, which were focused on balanced designs, unbalanced designs, and applications with many variables, where the multilevel structure is not of immediate interest in the substantive analyses.

Overall, our findings indicate that an AGM approach based on single-level MI could be a useful alternative to multilevel MI, particularly in challenging situations where multilevel MI can be less reliable. Specifically, in the less challenging situations, we found that multilevel MI generally performed well and that the AGM approach provided results that were similar to – but also not better than – those in multilevel MI. This was also true in unbalanced data, where the extended specification of the AGM approach provided results similar to multilevel MI. However, in the more challenging situations, the AGM approach often provided substantially more accurate results and was much less prone to computational problems than multilevel MI. By comparison, the GM approach appeared to have poorer theoretical properties and tended to perform worse than the AGM approach in most situations.

Despite these encouraging results, there are multiple limitations worth highlighting. First, we considered only multilevel designs with a two-level structure. The GM and AGM approaches could also be extended to higher-level or non-hierarchical structures. In fact, the AGM approach has already been proposed for handling missing data in cross-classified designs (Grund et al., 2023). However, research on these applications is still scarce, and future research should consider other design types in more detail, including three-level, cross-classified, and multiple-membership designs (see also Wijesuriya et al., 2020; Kadengye et al., 2014). Second, we only considered applications with continuous data. In principle, the GM and AGM approaches could also be combined with single-level imputation methods for categorical or ordinal data (e.g., Austin and van Buuren, 2023), but the use of (adjusted) group means might not be straightforward in these situations (e.g., with unordered categorical data). Third, although our simulations included conditions with many variables, the number of variables that we considered was still lower than in many real-world applications. Recent research has indicated that problems with many variables can be addressed in the FCS approach by combining conventional imputation methods with dimension reduction techniques (Audigier et al., 2016; Costantini et al., 2024; Grund et al., 2024; Howard et al., 2015). However, it is currently an open question whether and how these techniques can be used in multilevel designs. Related to this, we did not consider conditions with many variables and unbalanced designs, which would be an interesting scenario, because the extensions needed to accommodate strongly imbalanced group sizes come at the cost of increasing the number of variables in the imputation model. Finally, throughout the article, we only considered applications that aimed at relatively simple single- and multilevel analyses. This was done deliberately, because we believe that these types of applications are extremely common in practice (e.g., McNeish et al., 2016; Cameron and Miller, 2015) and remain under-explored in the missing data literature. Nonetheless, more complex analyses are also common, for example, when researchers employ multilevel analyses with random slopes and nonlinear effects, and the beneficial properties of the AGM approach may not transfer to these applications (see also Enders et al., 2020; Erler et al., 2016, 2017; Grund et al., 2021; Lüdtke et al., 2020; Keller and Enders, 2023).

To summarize, the aim of the present article was to explore a group-mean-based approach to multilevel MI, in which single-level MI approaches are augmented with (adjusted) group means to accommodate the multilevel structure of the data. Our findings indicate this approach may be a promising method for handling missing data in multilevel designs, which could be particularly useful in challenging situations, in which conventional methods perform less reliably, and in which the multilevel structure does not feature strongly in the intended analyses. We hope that future research will continue in this line of research and that researchers will find this approach useful for handling missing data in multilevel designs.

## Author Note

All additional files concerning this manuscript, including code, data, and the supplemental materials, are available at: https://osf.io/mpfzu/. A copy of this manuscript has been posted on the PsyArXiv preprint server: (Grund et al., 2025)

## Open Practices Statement

All additional files concerning this article, including the computer code and results of the simulation studies, the computer code and data for the example analysis, and the supplemental materials, are available at: https://osf.io/mpfzu/. None of the simulation studies reported in this article were preregistered.

## Data Availability

The simulation results and the simulated data for the example analysis are available on the OSF platform (https://osf.io/mpfzu/).

## Appendix: Bias in the GM and AGM Approaches

This appendix presents the derivation of the asymptotic bias of the between- and within-group variances in the GM and AGM approaches. We assume that the data have a balanced two-level structure, where a variable *Y* takes on values $Y_{ij}$ for person *i* ($i = 1, \ldots , n$) in group *j* ($j = 1, \ldots , J$) with

A1 $$\begin{aligned} Y_{ij} = \mu + \upsilon _j + \epsilon _{ij} \; , \end{aligned}$$

where $\mu$ is the grand mean, and $\upsilon _j$ and $\epsilon _{ij}$ are residuals with $\textit{Var}(\upsilon _j) = \tau ^2$ and $\textit{Var}(\epsilon _{ij}) = \sigma ^2$, respectively. We assume that the data are MCAR with $n_\textit{mis}$ persons with missing values and $n_\textit{obs}$ persons with observed values in each group, respectively, with $p = n_\textit{mis}/n$. We also assume that the first $n_\textit{obs}$ values per group are observed and that the number of groups approaches infinity ($J \rightarrow \infty$). The imputed values are denoted by $Z_{ij}$. If $Y_{ij}$ is observed, then $Z_{ij} = Y_{ij}$. Finally, we assume that the (adjusted) group means are computed based on only the observed values as described in the main text.

### GM approach

In the GM approach, the imputed values for *Y* are generated on the basis of the group means $\bar{Y}_{\bullet j}$ with the following model:

A2 $$\begin{aligned} Y_{ij} = \gamma \bar{Y}_{\bullet j} + e_{ij} \; , \end{aligned}$$

where $\gamma$ is a regression coefficient, and $e_{ij}$ are residuals with $\textit{Var}(e_{ij}) = \sigma _e^2$. The parameters are estimated from the observed data, and the imputed values are drawn from the posterior predictive distribution of the missing data. The expected values of $\hat{\gamma }$ and $\hat{\sigma }_e^2$ are given by

A3 $$\begin{aligned} E(\hat{\gamma })= &  \frac{Cov(Y_{ij}, \bar{Y}_{\bullet j})}{Var(\bar{Y}_{\bullet j})} = \frac{Cov(\upsilon _j + \epsilon _{ij}, \upsilon _j + \bar{\epsilon }_{\bullet j})}{Var(\upsilon _j + \bar{\epsilon }_{\bullet j})}\nonumber \\= &  \frac{\tau ^2 + \frac{1}{n_\textit{obs}} \sigma ^2}{\tau ^2 + \frac{1}{n_\textit{obs}} \sigma ^2} = 1\nonumber \\ E(\hat{\sigma }_e^2)= &  \frac{1}{n_\textit{obs} J - 2} \sum _{j=1}^{J} \sum _{i=1}^{n_\textit{obs}} E \Big [ (Y_{ij} - \hat{\gamma } \bar{Y}_{\bullet j})^2\Big ]\nonumber \\= &  \frac{1}{n_\textit{obs} J - 2} \sum _{j=1}^{J} \sum _{i=1}^{n_\textit{obs}} \left( 1 - \frac{1}{n_\textit{obs}} \right) \sigma ^2 \; . \end{aligned}$$

In the case with a large number of groups ($J \rightarrow \infty$), the expected value of $\hat{\sigma }_e^2$ approaches $E(\hat{\sigma }_e^2) = (1 - \frac{1}{n_\textit{obs}}) \sigma ^2$, and – as the posterior variances approach zero – the posterior values of the parameters approximately equal their expected values. The imputed values are then generated as

A4 $$\begin{aligned} Z_{ij}= \bar{Y}_{\bullet j} + e_{ij} \, , \quad \textit{Var}(e_{ij}) = \left( 1 - \frac{1}{n_\textit{obs}} \right) \sigma ^2 \; . \end{aligned}$$

The within-group variance $\sigma ^2$ can be estimated using the observed within-group variance (Snijders and Bosker, 2012)

A5 $$\begin{aligned} S_\textit{within}^2 = \frac{1}{J(n-1)} \sum _{j=1}^{J} \sum _{i=1}^{n} (Z_{ij} - \bar{Z}_{\bullet j})^2 \; . \end{aligned}$$

To obtain the bias of $S_\textit{within}^2$, the contributions of both the cases with observed data and the cases with imputed data must be considered. We begin with the group means in the completed data, which can be written as:

A6 $$\begin{aligned} \bar{Z}_{\bullet j} = \upsilon _j \!+\! \frac{1}{n_\textit{obs}} \sum _{i=1}^{n_\textit{obs}} \epsilon _{ij} \!+\! \frac{1}{n} \sum _{i= n_\textit{obs} \!+\! 1}^{n} e_{ij} = \bar{Y}_{\bullet j} + \frac{1}{n} \sum _{i= n_\textit{obs} + 1}^{n} e_{ij} \end{aligned}$$

For the cases with observed data, we can then write:

A7 $$\begin{aligned} \begin{aligned} E \left[ (Y_{ij} - \bar{Z}_{\bullet j})^2 \right]&= E \left[ \left( \upsilon _j + \epsilon _{ij} - \upsilon _j - \frac{1}{n_\textit{obs}} \sum _{i=1}^{n_\textit{obs}} \epsilon _{ij} - \frac{1}{n} \sum _{i=n_\textit{obs} + 1}^{n} e_{ij} \right) ^2 \right] \\&= E \left[ \left( \frac{n_\textit{obs} - 1}{n_\textit{obs}} \epsilon _{ij} - \frac{1}{n_\textit{obs}} \sum _{i' \! \ne i} \epsilon _{i' \! j} - \frac{1}{n} \sum _{i=n_\textit{obs} + 1}^{n} e_{ij} \right) ^2 \right] \\&= \left( 1 - \frac{1}{n_\textit{obs}} \right) \bigg ( 1 + \frac{n_\textit{mis}}{n^2} \bigg ) \, \sigma ^2 \end{aligned} \end{aligned}$$

For the cases with imputed data:

A8 $$\begin{aligned} E \left[ (Z_{ij} - \bar{Z}_{ \bullet j})^2 \right]&= E \left[ \left( \bar{Y}_{ \bullet j} + e_{ij} - \bar{Y}_{ \bullet j} - \frac{1}{n} \sum _{i=n_\textit{obs} + 1}^{n} e_{ij} \right) ^2 \right] \nonumber \\&= \left( 1 - \frac{1}{n_\textit{obs}} \right) \left( \frac{n-1}{n} - \frac{n_\textit{obs}}{n^2} \right) \sigma ^2 \end{aligned}$$

Combining Eqs. A7 and A8 gives the expected total deviation for all cases in group *j*:

A9 $$\begin{aligned} \sum _{i=1}^{n} E \left[ (Z_{ij} \!-\! \bar{Z}_{ \bullet j})^2 \right] \!=\! \left[ 1 \!-\! p \left( \frac{1}{n-1} \!+\! \frac{1}{n_\textit{obs}} \right) \right] (n-1) \sigma ^2 \; . \end{aligned}$$

The expected value of $S_\textit{within}^2$ is then given by

A10 $$\begin{aligned} E(S^2_\textit{within}) = \sigma ^2 - p \frac{n_\textit{obs} + n - 1}{(n-1) n_\textit{obs}} \sigma ^2 \; , \end{aligned}$$

and the bias of the estimator of the within-group variance can be calculated as

A11 $$\begin{aligned} \textit{Bias}(\hat{\sigma }^2) = E(S^2_\textit{within}) - \sigma ^2 = {} - p \frac{n_\textit{obs} + n - 1}{(n-1) n_\textit{obs}} \sigma ^2 \; . \end{aligned}$$

For the estimator of the between-group variance $\tau ^2$, we begin with the observed between-group variance (Snijders and Bosker, 2012) in the completed data:

A12 $$\begin{aligned} S^2_\textit{between} = \frac{1}{J-1} \sum _{j=1}^{J} (\bar{Z}_{\bullet j} - Z_{\bullet \bullet })^2 \; , \end{aligned}$$

To calculate the expected value of $S^2_\textit{between}$, we use the following relationship:

A13 $$\begin{aligned} E \left[ (\bar{Z}_{\bullet j} - \bar{Z}_{\bullet \bullet }) \right] ^2&= E \Bigg [ \Bigg ( \frac{J-1}{J} \bar{Z}_{\bullet j} - \frac{1}{J} \sum _{j' \! \ne j}^{} \bar{Z}_{\bullet j'} \Bigg ) \Bigg ]^2 \nonumber \\&= \frac{J-1}{J} E \left[ \bar{Z}_{\bullet j}^2 \right] \; . \end{aligned}$$

Using Eqs. A6 and A13, the expected value of $S^2_\textit{between}$ is then given by

A14 $$\begin{aligned} E(S^2_\textit{between}) = \tau ^2 + \frac{1}{n} \sigma ^2 + p \left[ \frac{1}{n} \left( 1-\frac{1}{n_\textit{obs}} \right) + \frac{1}{n_\textit{obs}} \right] \sigma ^2 \; . \end{aligned}$$

The bias of the estimator of the between-group variance can then be calculated as

A15 $$\begin{aligned} \textit{Bias}(\hat{\tau }^2) \!=\! E \left( S_\textit{between}^2 \!-\! \frac{S_\textit{within}^2}{n} \right) \!-\! \tau ^2 \!=\! p \frac{n_\textit{obs} + n - 1}{(n-1) n_\textit{obs}} \sigma ^2 \; . \end{aligned}$$

### AGM approach

In the AGM approach, the imputed values are generated on the basis of the adjusted group means $\tilde{Y}_{ij}$. Reusing some notation, the imputations are generated with the following model:

A16 $$\begin{aligned} Y_{ij} = \gamma \tilde{Y}_{ij} + e_{ij} \; , \end{aligned}$$

where $\gamma$ is a regression coefficient, and the $e_{ij}$ residuals with $\textit{Var}(e_{ij}) = \sigma _e^2$. The parameters are again estimated from the observed data with

A17 $$\begin{aligned} \begin{aligned} E(\hat{\gamma })&= \frac{Cov(Y_{ij}, \tilde{Y}_{ij})}{Var(\tilde{Y}_{ij})} = \frac{ Cov(\upsilon _j + \epsilon _{ij}, \upsilon _j + \frac{1}{n_\textit{obs}-1} \sum _{i' \! \ne i} \epsilon _{i' \! j}) }{ Var(\upsilon _j + \frac{1}{n_\textit{obs}-1} \sum _{i' \! \ne i} \epsilon _{i' \! j}) }\\&= \frac{\tau ^2}{\tau ^2 + \frac{1}{n_\textit{obs} - 1} \sigma ^2} \\ E(\hat{\sigma }_e^2)&= \frac{1}{n_\textit{obs} J - 2} \sum _{j=1}^{J} \sum _{i=1}^{n_\textit{obs}} E \Big [ (Y_{ij} - \hat{\gamma } \tilde{Y}_{ij})^2\Big ]\\&= \frac{1}{n_\textit{obs} J - 2} \sum _{j=1}^{J} \sum _{i=1}^{n_\textit{obs}} {\left( 1 + \frac{1}{n_\textit{obs}-1} E(\hat{\gamma }) \right) }\sigma ^2 \; . \end{aligned} \end{aligned}$$

For the case with $J \rightarrow \infty$, we write $\gamma = \frac{\tau ^2}{\tau ^2 + \sigma ^2 / (n_\textit{obs} - 1)}$. The expected value of $\hat{\sigma }_e^2$ then approaches $E(\hat{\sigma }_e^2) = \big ( 1 + \frac{1}{n_\textit{obs}-1} \gamma \big ) \sigma ^2$, and the imputed values are generated as:

A18 $$\begin{aligned} Z_{ij}= \gamma \tilde{Y}_{ij} + e_{ij} \, , \quad \textit{Var}(e_{ij}) = {\left( 1 + \frac{1}{n_\textit{obs}-1} \gamma \right) }\sigma ^2 \; . \end{aligned}$$

To derive the bias of $S_\textit{within}^2$, we again begin with the group means in the completed data and define $c = \big ( 1 + \frac{n_\textit{mis}}{n_\textit{obs}} \gamma \big )$. The group means can then be written as:

A19 $$\begin{aligned} \begin{aligned} \bar{Z}_{\bullet j}&= \frac{n_\textit{obs} c}{n} \upsilon _j + \frac{c}{n} \sum _{i=1}^{n_\textit{obs}} \epsilon _{ij} + \frac{1}{n} \sum _{i=n_\textit{obs}+1}^{n} e_{ij}\\&= \frac{n_\textit{obs} c}{n} \bar{Y}_{\bullet j} + \frac{1}{n} \sum _{i=n_\textit{obs}+1}^{n} e_{ij} \end{aligned} \; . \end{aligned}$$

For the cases with observed data, we can then write:

A20 $$\begin{aligned} E\! \left[ (Y_{ij} - \bar{Z}_{\bullet j})^2 \right] \!= &  \! E \left[ \left( \upsilon _j + \epsilon _{ij} - \frac{n_\textit{obs} c}{n} \upsilon _j \right. \right. \nonumber \\ &  \! \left. \left. - \frac{c}{n} \sum _{i=1}^{n_\textit{obs}} \epsilon _{ij} - \frac{1}{n} \sum _{i=n_\textit{obs}+1}^{n} e_{ij} \right) ^2 \right] \nonumber \\= &  \! E \left[ \left( \frac{n_\textit{mis}}{n} (1-\gamma ) \upsilon _j + \frac{n - c}{n} \epsilon _{ij}\right. \right. \nonumber \\ &  \! \left. \left. - \frac{c}{n} \sum _{i' \! \ne i} \epsilon _{i' \! j} - \frac{1}{n} \sum _{i=n_\textit{obs}+1}^{n} e_{ij} \right) ^2 \right] \\= &  \! \frac{n_\textit{mis}^2}{n^2} (1-\gamma )^2 \tau ^2 \nonumber \\ &  + \frac{(n - c)^2}{n^2} \sigma ^2 + \frac{(n_\textit{obs}-1) c^2}{n^2} \sigma ^2\nonumber \\ &  + \frac{n_\textit{mis}}{n^2} {\left( 1 + \frac{1}{n_\textit{obs}-1} \gamma \right) }\sigma ^2 \; . \nonumber \end{aligned}$$

For the cases with imputed data:

A21 $$\begin{aligned} E\! \left[ (Z_{ij}\! -\! \bar{Z}_{\bullet j})^2 \right] \!&=\! E \left[ \! \left( \gamma \bar{Y}_{\bullet j}\! +\! e_{ij}\! -\! \frac{n_\textit{obs} c}{n} \bar{Y}_{\bullet j}\! -\! \frac{1}{n} \sum _{i=n_\textit{obs}+1}^{n} e_{ij} \right) ^2 \right] \nonumber \\&=\! E \left[ \!\left( \! - \frac{n_\textit{obs}}{n} (1\! -\! \gamma ) \bar{Y}_{\bullet j}\! +\! \frac{n\!-\!1}{n} e_{ij}\! -\! \frac{1}{n} \sum _{i' \! \ne i} e_{i' \! j} \right) ^2 \right] \\&\!=\! \frac{n_\textit{obs}^2}{n^2} (1 \!-\! \gamma )^2 \tau ^2 \!+\! \frac{n_\textit{obs}}{n^2} \left[ (1 \!-\! \gamma )^2 \!-\! {\left( 1 + \frac{1}{n_\textit{obs}-1} \gamma \right) }\right] \sigma ^2\nonumber \\&\quad + \frac{n-1}{n} {\left( 1 + \frac{1}{n_\textit{obs}-1} \gamma \right) }\sigma ^2 \nonumber \; . \end{aligned}$$

Combining these equations, the expected value of $S_\textit{within}^2$ is now given by

A22 $$\begin{aligned} E(S^2_\textit{within}) \!=\! \sigma ^2 + p \frac{n_\textit{mis} - \gamma + 1}{(n - 1) (n_\textit{obs} - 1)} \gamma \sigma ^2 \; . \end{aligned}$$

The bias of the estimator of the within-group variance is then equal to

A23 $$\begin{aligned} \textit{Bias}(\hat{\sigma }^2) = E(S^2_\textit{within}) - \sigma ^2 = p \frac{n_\textit{mis} - \gamma + 1}{(n - 1) (n_\textit{obs} - 1)} \gamma \sigma ^2 \; . \end{aligned}$$

Using Eqs. A19 and A13, the expected value of $S^2_\textit{between}$ is equal to

A24 $$\begin{aligned} E(S^2_\textit{between}) \!=\! \tau ^2 + \frac{1}{n} \left[ c^2 (1-p) + p \frac{(n_\textit{obs} - 1) (1 - \gamma ) - (n + n_\textit{mis} \gamma ) \gamma }{n_\textit{obs} - 1} \right] \sigma ^2 \; . \end{aligned}$$

The bias of the estimator of the between-group variance can then be calculated as

A25 $$\begin{aligned} \textit{Bias}(\hat{\tau }^2)= &  E \left( S_\textit{between}^2 - \frac{S_\textit{within}^2}{n} \right) - \tau ^2\nonumber \\= &  {} - p \frac{n_\textit{mis} + \frac{n_\textit{mis}-1}{n_\textit{obs}} \gamma + 1}{(n-1)(n_\textit{obs}-1)} \gamma \sigma ^2 \; . \end{aligned}$$

## Footnotes

1 In principle, the inclusion of the group sizes could also have adverse effects, because it increases the collinearity between the terms in the imputation models. However, previous studies have not revealed any meaningful impact of collinearity even in weakly imbalanced designs, where the collinearity would be strongest.

2 To investigate the potential differences between MCAR and MAR data, we replicated a subset of the conditions from Study 1 with MAR data. The results were very similar – except for LD, which yielded strongly biased results for most of the parameters – and can be found in Supplement B in the supplemental online materials.

3 Given the balanced design in Study 1, the true values of the parameters in the manifest covariate models could also have been computed from the population parameters. However, as this is no longer true in more general settings with unbalanced designs (Study 2), we decided to use the average values under CD throughout to make the results comparable across studies.

4 The failed replications were due to computational errors returned by the lme4 package Bates et al. 2015, on which 2l.continuous and other implementations of multilevel MI are based. The errors were not deterministic but tended to occur within a small number of iterations in the FCS algorithm.

## Declarations

### Code availability

The computer code for the simulations is available on the OSF platform (https://osf.io/mpfzu/).
